# Time-resolved imaging of electron beam powder bed fusion using an X-ray microscope optimized for white beam radiation

**DOI:** 10.1107/S1600577525010057

**Published:** 2026-01-01

**Authors:** Pidassa Bidola, Nick Semjatov, Gabriel Spartacus, Hans-Henrik König, Guilherme Abreu-Faria, Johannes Klingenberg, Jens Brehling, Christina Krywka, Peter Staron, Greta Lindwall, Carolin Körner, Chrysoula Ioannidou, Felix Beckmann

**Affiliations:** a Institute of Materials Physics Helmholtz-Zentrum Hereon Max-Planck-Straße 1 21502Geesthacht Germany; b Chair of Materials Science and Engineering for Metals Friedrich-Alexander-Universität Erlangen-Nürnberg Martensstraße 5 91058Erlangen Germany; c Department of Materials Science and Engineering KTH Royal Institute of Technology Brinellvägen 23 SE-10044Stockholm Sweden; Tohoku University, Japan

**Keywords:** high-speed X-ray imaging, electron beam powder bed fusion, synchrotron white beam, crack evolution

## Abstract

A high-speed synchrotron radiography system has been developed to facilitate *in situ* imaging of dynamic processes in electron beam powder bed fusion (PBF-EB). Using the P61A White Beam Engineering Materials Science beamline at PETRA III, this system achieves high temporal resolution and a spatial resolution of approximately 10 µm. The scintillator screens are coupled to a diamond plate and housed within a specialized nitro­gen gas cooling system, effectively mitigating thermal stress caused by the intense synchrotron beam. These innovative components ensure stable imaging performance and enhance the system’s ability to operate under extreme conditions. By resolving fringes at short propagation distances for the partially coherent beam, the imaging system has enabled the efficient visualization of crack formation and pore evolution in high-*Z* materials, such as nickel-based superalloys, during the PBF-EB process. These advances not only optimize imaging in extreme environments but also open new avenues for high-energy synchrotron applications, including dynamic phase imaging and laser welding studies of dense samples.

## Abstract 2

A high-speed X-ray microscope design optimized for white beam synchrotron radiation enables real-time *in situ* imaging of crack dynamics during electron beam powder bed fusion (PBF-EB) of high-*Z* materials. With high temporal resolution and 10 µm spatial resolution, the system reveals previously unobservable crack evolution behavior in Ni-based superalloys, potentially advancing understanding of additive manufacturing processes.

## 1. Introduction

Advances in high-speed X-ray imaging have significantly enhanced our ability to study dynamic processes in materials science, particularly in additive manufacturing (AM) techniques like electron beam powder bed fusion (PBF-EB). PBF-EB is distinguished by its use of an electron beam as a heat source, enabling ultra-high scanning speeds (∼10 km s^−1^; Körner, 2016 ▸) and processing at extremely high temperatures (>1100°C; Fu & Körner, 2022 ▸) in a vacuum environment which prevents material oxidation. These characteristics make PBF-EB ideal for processing crack-sensitive materials such as high γ′ Ni-based superalloys, titanium aluminides (used in turbine blade production) and various refractory alloys. Given its advantages, there is strong interest in understanding the mechanisms of cracking during PBF-EB processes to optimize processing conditions and guide alloy development for improved manufacturability.

However, many crack-sensitive and high-temperature materials include refractory elements which have low atomic diffusivity and high X-ray absorption. These properties, combined with the need to image substantial material volumes to replicate industrial conditions, create significant challenges using conventional synchrotron imaging techniques to track the rapid dynamics of the PBF-EB process.

High-speed X-ray imaging makes use of an extremely high photon flux to achieve high frame rates and short exposure times. For dynamic imaging, the scintillator must provide a high light yield while withstanding the intense X-ray flux such as that available from a synchrotron source. Owing to their broadband (polychromatic) spectrum, synchrotron white X-ray beams (Kastengren, 2019 ▸) provide very high photon flux, making them highly effective for additive manufacturing studies and facilitating time-resolved X-ray imaging and diffraction (Wu *et al.*, 2021 ▸; Chen *et al.*, 2021 ▸; Zhao *et al.*, 2017 ▸; Escano *et al.*, 2022 ▸; Di Michiel *et al.*, 2005 ▸). Early pioneering work has already demonstrated the feasibility of high-speed synchrotron radiography and tomography (Di Michiel *et al.*, 2005 ▸; Rack *et al.*, 2010 ▸; Wang *et al.*, 2008 ▸), which has since been extended to increasingly challenging material processes.

When exposed to synchrotron white X-ray beams, fast imaging X-ray devices can suffer radiation damage. Only a small fraction of the X-ray energy absorbed by scintillators is converted into visible light, and these scintillators are delicate single-crystal plates sensitive to temperature changes (Yanagida *et al.*, 2013 ▸). Excessive radiation can cause thermal stress and potentially crack the scintillator (Kastengren, 2019 ▸). High radiation flux can also damage lenses (Di Michiel *et al.*, 2005 ▸) and other optical components in the beam path. A suitable setup (Di Michiel *et al.*, 2005 ▸; Matusik *et al.*, 2018 ▸) is essential to prevent scattered high-energy radiation from hitting the camera sensor, which can create white spots (zingers) that distort images (Zhou *et al.*, 2018 ▸). Additionally, scintillators charged by ionizing radiation can attract dust, complicating flat-field correction. High radiation doses may also pose ozone hazards, which can be tracked with ozone sensors (Agrawal *et al.*, 2021 ▸; Koch, 1994 ▸).

In-depth research on the interaction between the electron beam and materials during PBF-EB is essential (Ioannidou *et al.*, 2022 ▸) and recent developments have attempted to address these challenges by enhancing imaging components, such as scintillators and cooling systems, to withstand extreme synchrotron conditions (Farla *et al.*, 2022 ▸; Bührer *et al.*, 2019 ▸; Olbinado *et al.*, 2017 ▸). Despite these advances, current systems still fall short of providing the resolution, speed and stability required for *in situ* studies of crack formation and pore evolution during PBF-EB processes. A fundamental understanding of these phenomena is critical for optimizing processing conditions and informing alloy design. In response to these limitations, we introduce here a custom-designed high-speed X-ray radiography system specifically developed on the P61A White Beam Engineering Materials Science beamline at the PETRA III storage ring of the Deutsches Elektronen-Synchrotron (DESY). This beamline, operated by Helmholtz-Zentrum Hereon, has achieved a dose rate in the hundreds of grays per second, with an effective measurement of 1 kGy s^−1^ recorded using a microdiamond chamber and a 20 mm copper absorber (Schültke *et al.*, 2022 ▸). We tackled the thermal problems that the scintillator may experience due to the high photon flux on the P61 beamline. The occurrence of zingers was notably minimized by employing a 25 mm thick lead housing (Wroblewski, 2015 ▸) and utilizing a stepped arrangement of reflective mirrors. Additionally, we implemented dedicated convective cooling with nitro­gen gas (Zhou *et al.*, 2018 ▸), supplied at a pressure of 1.5 bar, and coupled the scintillator to a diamond plate, which facilitated a quicker achievement of steady state and allowed for a gradual decline in light output over time. This approach promotes stable image quality during high-flux operation. Consequently, we were able to achieve a resolution of approximately 10 µm, determined using a slanted edge. A frame rate of 15 kHz was utilized for initial radiographic recordings during electron beam remelting experiments of bulk CMSX-4 samples, proving that Ni-based superalloys with high concentrations of high-*Z* elements could be imaged (König *et al.*, 2023 ▸). The system has demonstrated the capability to resolve fringes at short propagation distances between 50 and 140 cm, enabling single-distance propagation-based phase contrast imaging (PB-PCI). By facilitating real-time monitoring of crack dynamics in thick high-*Z* materials, this system provides a notable step forward in enabling *in situ* studies of PBF-EB processes under specific high-flux conditions.

This work demonstrates the application of the developed system to *in situ* imaging of the PBF-EB process with CMSX-4, a second-generation Ni-based superalloy with a high X-ray absorptivity. Through detailed observations of crack formation and evolution, we validate the system’s ability to overcome key challenges in synchrotron imaging. The system design and findings also offer useful insights that could inform the development of future high-speed imaging setups for related applications such as phase contrast imaging or dynamic studies like laser welding and cavity collapse. By addressing key challenges in high-speed X-ray imaging under extreme flux conditions, this study contributes to advancing dynamic imaging capabilities for additive manufacturing research.

## 2. Microscope design

### 2.1. Camera

Ultrafast X-ray microscopy at third-generation synchrotrons, such as PETRA III, benefits from recent advances in high-resolution CMOS (complementary metal–oxide–semiconductor) sensors (Leung *et al.*, 2018 ▸; Parab *et al.*, 2018 ▸; Kaufmann *et al.*, 2023 ▸). A phantom v2640 ultrahigh-speed camera from Vision Research, USA, is deployed, with a full frame size of 2048 × 1952 pixels and a physical pixel size of 13.5 µm. In high-speed mode (HS) and full resolution, a recording of 6600 frames per second is achieved. When binned, a frame rate of 25 kHz can be reached using the entire field of view (FoV). Higher frame rates could also be used, allowing a minimum exposure time of down to 142 ns, by cropping the FoV.

### 2.2. Optics

**Figure 1:**
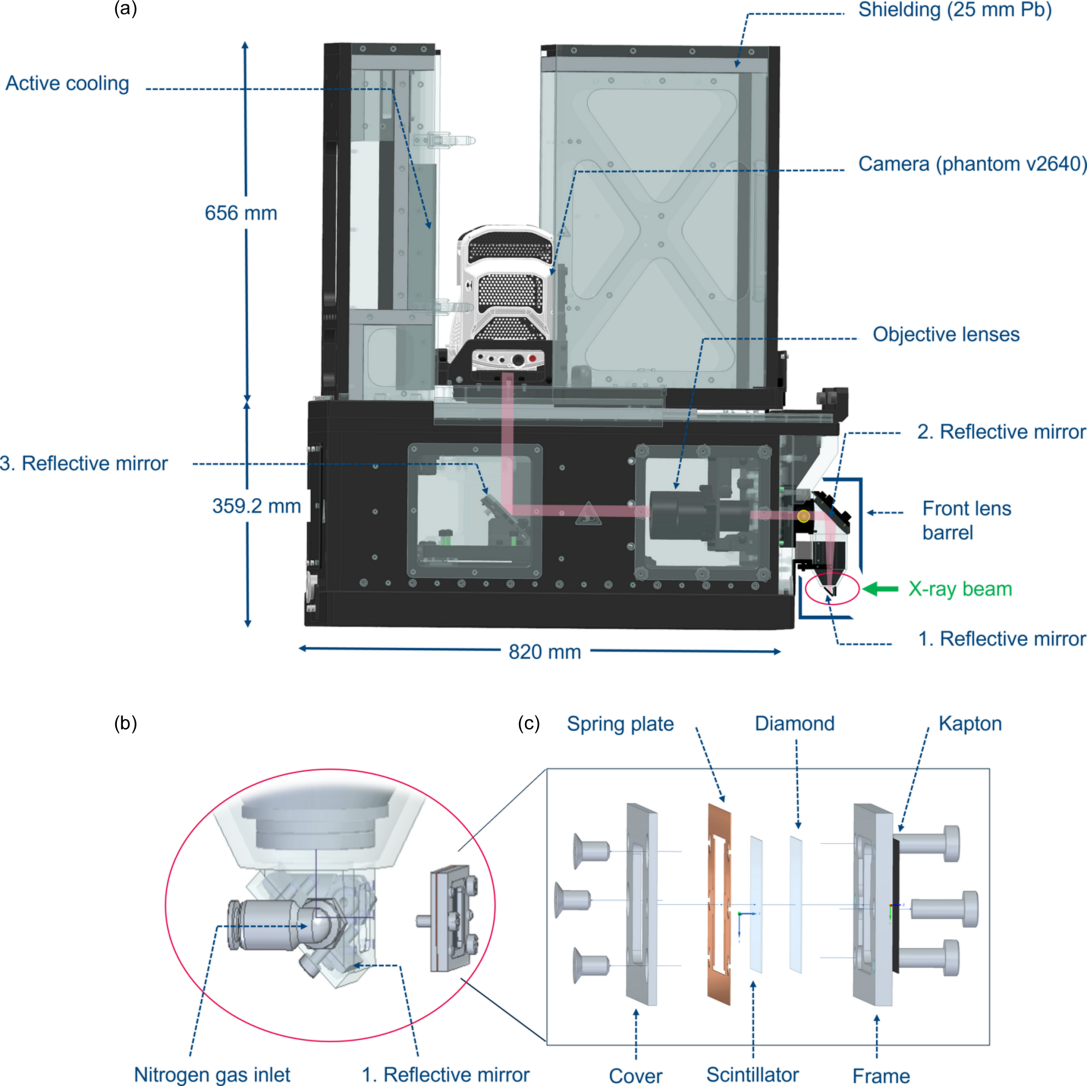
(*a*) Overview of the X-ray imaging microscope. The visible light (highlighted in pink), resulting from the white X-ray beam converted by the scintillator, is guided to the sensor via reflection mirrors. The housing of the camera is equipped with a cooling device. (*b*) A stream of nitro­gen gas feeds the scintillator through an inlet and outlet. (*c*) A stable mounting of the scintillator holder assembly prevents vibrations. A 100 µm thin diamond plate is coupled to the scintillator via direct contact, enabling more efficient dissipation of heat generated during X-ray interactions.

The microscope comprises two reflecting mirrors on either side of a motorized group of focusing lenses that direct the visible light from the scintillator to the magnifying lenses. These components are housed in an assembly known as the front lens barrel [Fig. 1 ▸(*a*)]. A third mirror directs the visible light from the selected 5× and 10× magnification lenses (numerical aperture NA = 0.25) to the high-speed camera. A back focal distance between the sensor and the lens can be repeated with high accuracy for each of the lenses. The step-shaped arrangement of the optical path prevents the camera sensor from being exposed to the direct beam (Xie *et al.*, 2019 ▸; Halls *et al.*, 2017 ▸) and allows sufficient distance between the sensor and the impact area of the white X-ray beam with the imaging system, minimizing scattering artifacts (Di Michiel *et al.*, 2005 ▸; Zhou *et al.*, 2018 ▸). The optical focus at the position of the camera sensor is adjusted by motorized displacement of the lens assembly within the front lens barrel [*e.g.* Singh *et al.* (2018 ▸)]. The optical components were custom designed by POG Präzisionsoptik Gera GmbH, Germany.

### 2.3. Shielding

Zingers are minimized by sufficient shielding near the optical setup to absorb scattered radiation before it reaches the CMOS chip. In our case, the use of ten wigglers as a synchrotron radiation source resulted in a high scattered radiation background, which required 2.5 cm thick lead shielding walls around the microscope to reduce the dose effectively (Bassey *et al.*, 2015 ▸; Wroblewski, 2015 ▸). We note that this level of shielding is specific to our high-flux synchrotron environment; in other experimental contexts such as high-speed imaging of laser welding, frame rates of 100 kHz to 10 MHz have been achieved with less sophisticated detector protection (Schmidt *et al.*, 2024 ▸).

### 2.4. Scintillator holder assembly

Whilst a nitro­gen gas stream removes dust particles from the scintillator surface (Zhou *et al.*, 2018 ▸), it was also used to provide the scintillator with sufficient cooling by employing nitro­gen gas supplied at a pressure of 1.5 bar (König *et al.*, 2023 ▸). This approach enables a steady flow past the surface of the scintillator with a small angle of attack, which protects the fragile scintillator from a force exerted towards the beam direction [Fig. 1 ▸(*b*), and Fig. S1 in the supporting information].

The scintillator is coupled [Fig. 1 ▸(*c*)] to a 100 µm polycrystalline diamond plate [processed via chemical vapor deposition (CVD)] to enhance heat dissipation (Kandlakunta *et al.*, 2019 ▸) from the aftermath of white X-ray beam inter­action.

### 2.5. Software

Proprietary software (*PCC*) from Vision Research controls the camera for recordings and review of the already acquired sequences. Recordings are stored in the proprietary cine format, storing the recorded data in 12-bit raw, as well as frame time stamps with nanosecond resolution. The camera is provided with a RAM of 72 GB that can be allocated to 63 partitions, thus allowing successive recordings each time a trigger signal is delivered.

A TTL (transistor–transistor logic) signal was provided in this work by the PBF-EB system to the dedicated port on the camera, therefore synchronizing the launch of the melting process and the start of the recording at the camera.

## 3. Experimental

### 3.1. Overview of the beamline

**Figure 2:**
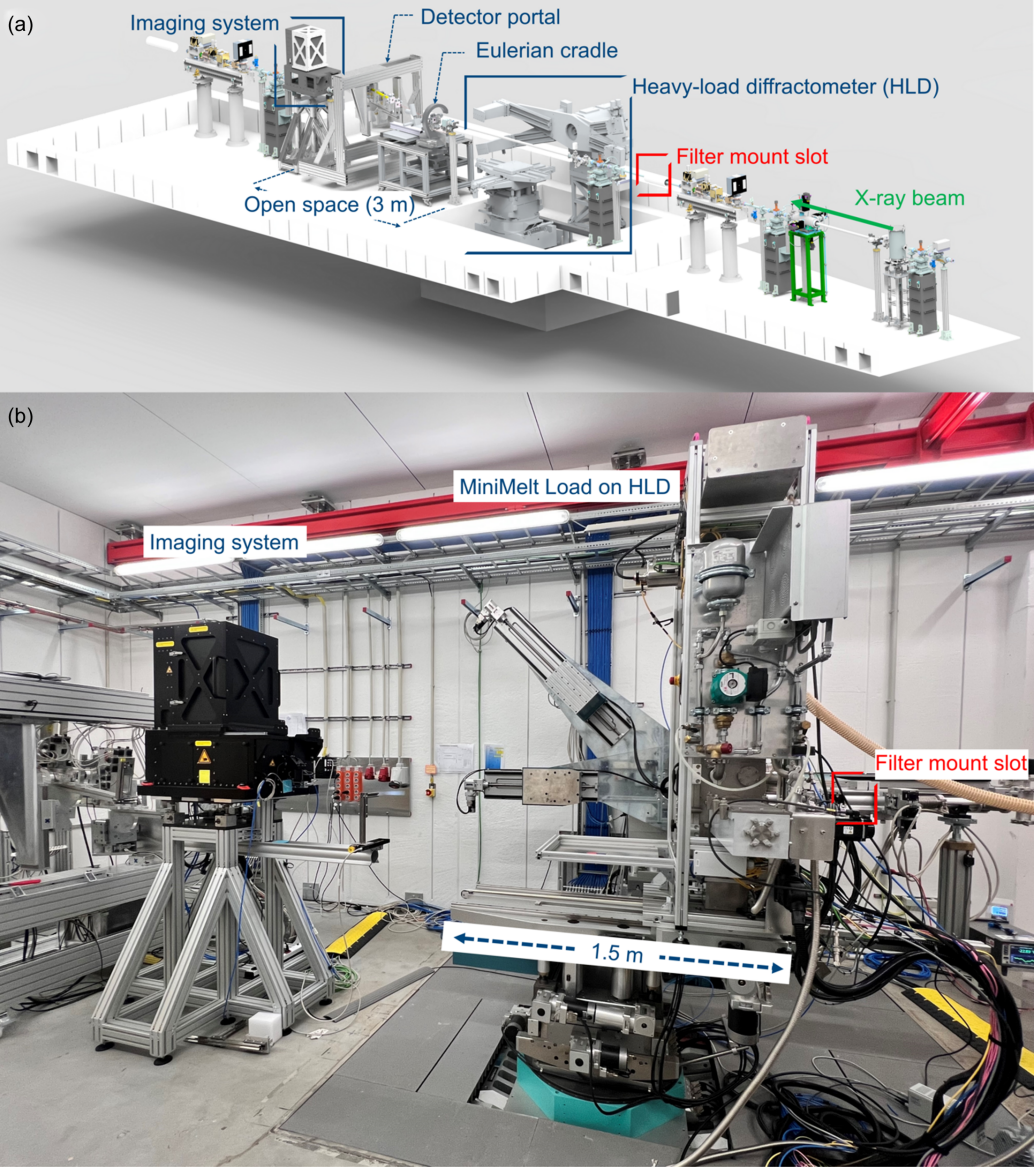
Overview of the experimental hutch (P61A at PETRA III). (*a*) Primary device for energy-dispersive diffraction and room for white X-ray imaging. (*b*) Photograph of the ‘MiniMelt’ PBF-EB machine as installed during the experiments. The slot for the filter holder is highlighted.

The polychromatic white X-ray beam is mainly used on the P61A station for energy-dispersive diffraction. The experimental hutch also has an Eulerian cradle for small samples, which are measured with a pair of detectors mounted in the detector portal. These two instruments are mobile and provide free experimental space for imaging of 3 m [Fig. 2 ▸(*a*)]. The station is equipped with a heavy-load diffractometer (HLD) that can support a mass of up to one tonne on which the PBF-EB sample environment ‘MiniMelt’ (König *et al.*, 2023 ▸) was placed during *in situ* imaging experiments. The translational motion on the HLD of about 1.5 m in the direction of the beam path is useful to determine the ideal X-ray propagation distance between the imaging system and the sample position inside the MiniMelt [Fig. 2 ▸(*b*)]. This ideal distance allows the camera to resolve the X-ray fringes and improve contrast.

### 3.2. Characterization of the setup

#### 3.2.1. Filters

A set of X-ray filters, cooled by convection using pressurized air, was installed upstream of the MiniMelt system (filter mount slot in Fig. 2 ▸) to decrease the beam flux reaching the instruments (Agrawal *et al.*, 2021 ▸; Halls *et al.*, 2017 ▸) and trim the energy spectrum that contributes to the image contrast (Zhou *et al.*, 2018 ▸).

**Figure 3:**
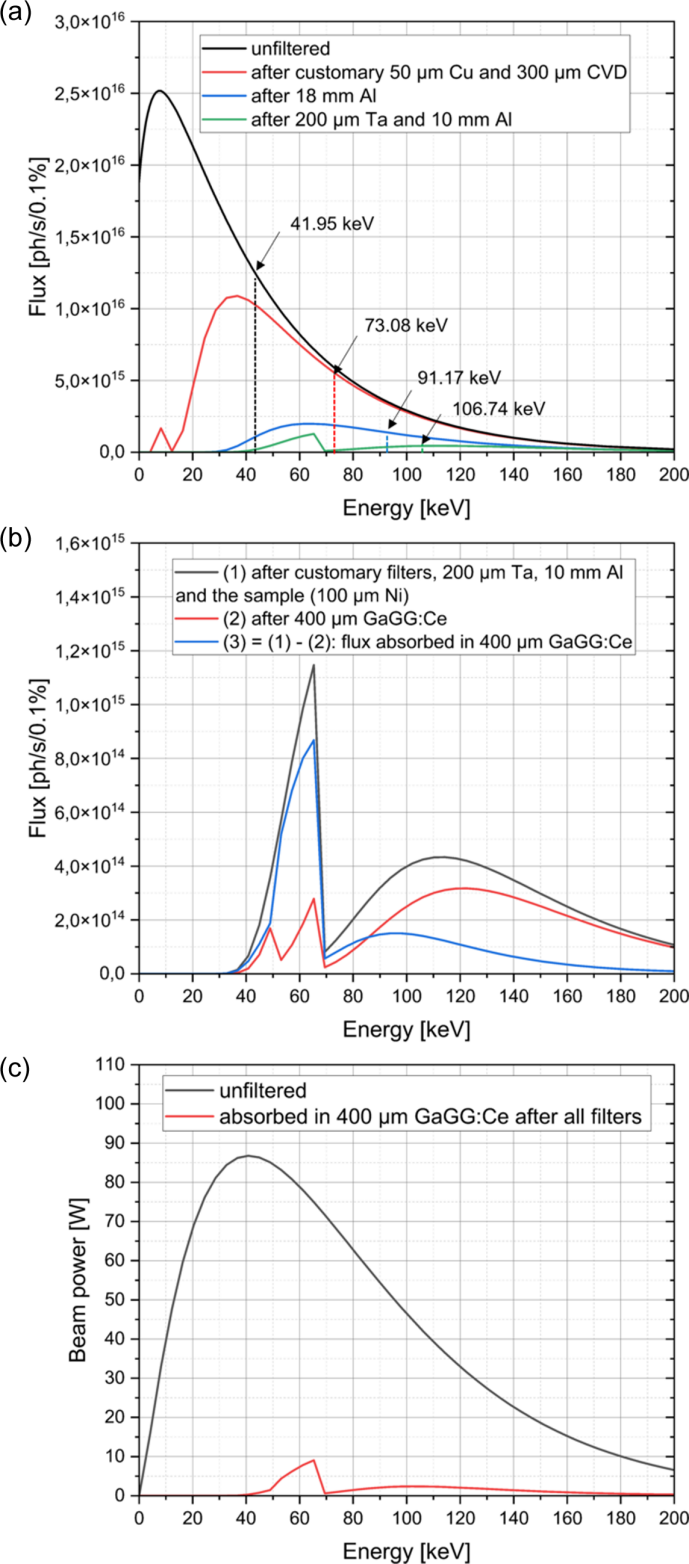
Influence of different absorbers on the transmitted X-ray flux, as simulated with *SPECTRA 11.0*. (*a*) The energy spectra after interaction of the radiation with several materials in the beam path and the resulting average energies are displayed. The diamond plate is labeled ‘CVD’. (*b*) Residual flux after high-pass filter materials, the PBF-EB machine (here considering a 100 µm thick nickel sample mounted inside) and a 400 µm thick GAGG:Ce scintillator. (*c*) The beam power calculated from the flux absorbed within the GaGG:Ce scintillator.

The absorption of the white X-ray beam by various filter materials was simulated using the software *SPECTRA 11.0* (Tanaka, 2021 ▸). Figs. 3 ▸(*a*) and 3 ▸(*b*) show the resulting spectrum when the X-ray beam interacts with any material in the beam path, including the sample inside the MiniMelt and the scintillator. During imaging experiments using the MiniMelt system, a powder hill with an inclination of 35°, a height of 2 mm and a top width of 1 mm is usually created to investigate the PBF-EB powder melting process, which represents the typically imaged sample geometry (König *et al.*, 2023 ▸). Although such geometries of the Ni-based powder bed could be realized during experiments (see Section 4.4), possible deviations from it had to be considered. We therefore assumed a minimum Ni powder bed/sample thickness of just 100 µm when simulating the critical X-ray flux above which mechanical damage of the scintillator would be expected. The material configuration of the MiniMelt is illustrated in Fig. S1. Except for aluminium (Al), mater­ials such as lead (Pb), platinum (Pt), tungsten (W) and tantalum (Ta) were used as filters during the simulation based on their atomic number and their ability to reduce the flux of high-energy X-rays. Nevertheless, their melting temperature is essential to withstand the heat load induced by the beam. Under the current experimental conditions, for example, the use of Pb is hindered by its melting during prolonged direct exposure to the high flux of white synchrotron radiation. In contrast, a combination of Al and Ta [Figs. 3 ▸(*a*) and 3 ▸(*b*)] proved effective during the experiments.

While the combination of different filters influences the spectrum, the required filter thickness was determined by comparing the simulated spectra with a reference thickness of Al. Therefore, an aluminium step wedge was used to identify the reference thickness at which the scintillator avoids fracture from heat-load-induced thermal stress.

**Figure 4:**
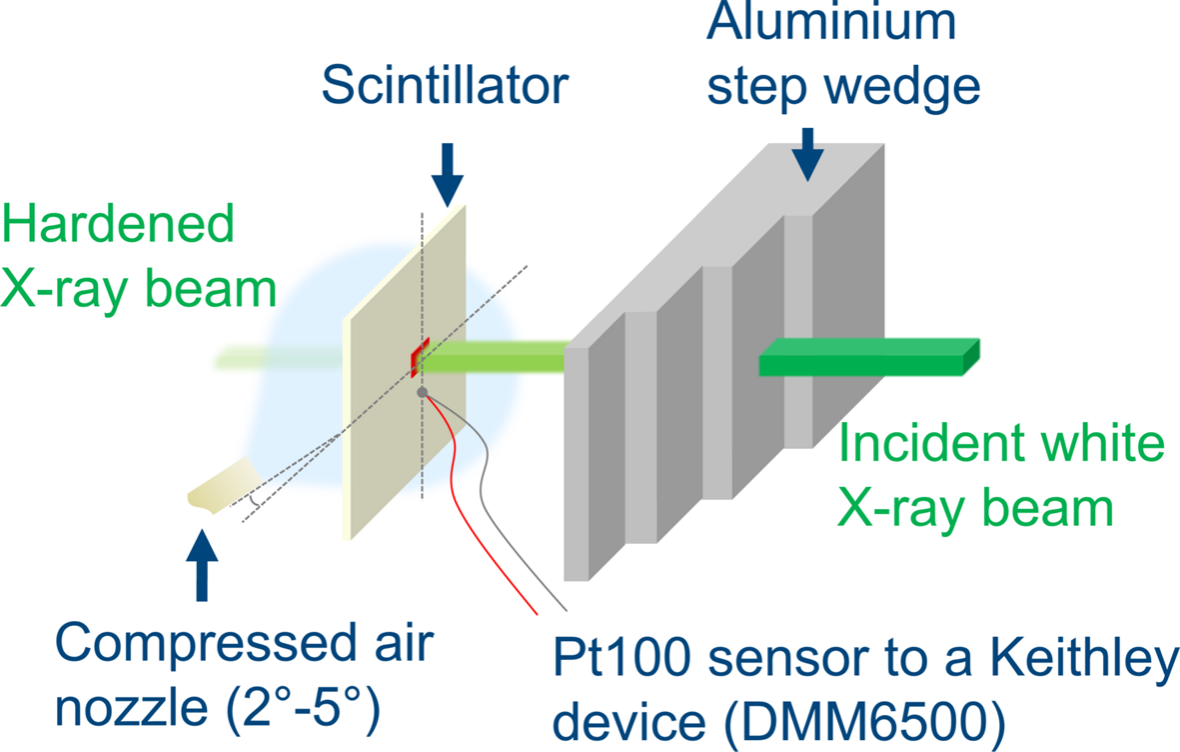
Experimental setup for the determination of (i) the efficiency of the convective cooling at the scintillator and (ii) the critical material thickness to avoid scintillator damage. The step wedge is shifted from thick to thin material until the scintillator starts to exhibit damage.

The experimental setup in Fig. 4 ▸ was used to demonstrate the efficiency of convective cooling and define the critical thickness of a filter material to protect the scintillator from damage.

A Keithley Pt100 sensor (DMM6500) was used to measure the temperature variations in the vicinity of the beam impinging on the scintillator. In this preliminary experiment, a stream of compressed air could be applied to the scintillator. Also, the aluminium step wedge was inserted into the beam path from the thickest (safe) step and moved to progressively thinner steps until scintillator failure occurred. A thickness of 18 mm of Al was determined as the minimum reference thickness at which no scintillator failure was observed under the given conditions with compressed air cooling.

By calculating the spectrum as a function of the thickness of the reference material before which the scintillator starts to exhibit damage, the limit in safely usable X-ray flux is identified. If the thickness of another filter or a combination of filters results in a lower spectral intensity [Fig. 3 ▸(*a*)], the scintillator is expected to be safe from radiation-induced damage. As an example, a combination of 10 mm Al and 200 µm Ta was identified and confirmed to be safe for operation without the risk of damaging the scintillator.

From the absorbed flux in the scintillator in Fig. 3 ▸(*b*), the spectrum of the beam power in a 400 µm thick GaGG:Ce scintillator is obtained by simple multiplication with the respective energies. A comparison with the raw beam power is displayed in Fig. 3 ▸(*c*). For reference, on the high-energy wiggler beamline (P61) ten wigglers produce a white beam delivering ∼121 W into a 1 mm × 1 mm spot at 40 m (∼121 W mm^−2^); with the mandatory 300 µm CVD diamond and 50 µm Cu plates, as well as additional attenuators (10 mm Al + 200 µm Ta), the power density was substantially reduced (although it was not further quantified here), providing conditions for stable operation of the scintillator without observable damage.

#### 3.2.2. Scintillators

The criteria for selecting scintillators for fast imaging are summarized as a high stopping power to absorb a significant fraction of the incident X-rays, a high light output to achieve acceptable signal-to-noise ratios and fast decay to avoid the accumulation of afterglow (Rutherford *et al.*, 2016 ▸; Olbinado *et al.*, 2017 ▸; Marton *et al.*, 2014 ▸; Luo *et al.*, 2012 ▸). In the work of Di Michiel *et al.* (2005 ▸), the ideal scintillators for high and low resolution were classified in the context of the experimental environment. Therefore, we investigated some commercially available fast-decaying scintillators for fast imaging at synchrotrons (*e.g.* CdW04, LuAG:Ce and GaGG:Ce) with regard to our experimental conditions.

**Figure 5:**
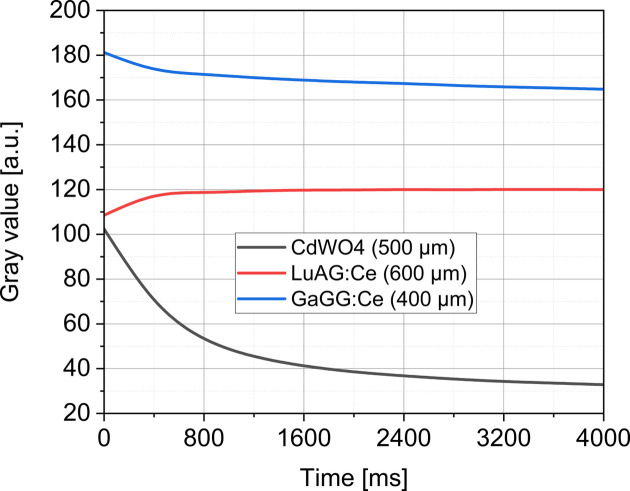
Temporal behavior of the light output of selected scintillators suitable for high-speed imaging, measured under constant white synchrotron X-ray flux with 200 µm Ta and 10 mm Al filters. Data points represent the average gray level recorded every 400 ms over a 2 mm × 1 mm FoV for CdWO_4_ (500 µm), LuAG:Ce (600 µm) and GaGG:Ce (400 µm) scintillators. All scintillators were vented according to our design and coupled to a diamond plate.

The dependence of the brightness of a scintillator on heat stress was emphasized by Yanagida *et al.* (2013 ▸), especially the occurrence of thermal quenching as a function of temperature. Therefore, we indirectly evaluated the behavior of the selected scintillators in the grayscale of the camera during white beam exposure. This experiment was performed with the scintillator mount described in Figs. 1 ▸(*b*) and 1 ▸(*c*). Fig. 5 ▸ shows the mean grayscale response curve measured every 400 ms on the selected scintillators in a FoV of 2 mm × 1 mm. The dark curve in Fig. 5 ▸ shows that CdWO_4_ is significantly altered by the radiation flux. GaGG:Ce is less impacted and provides better light output than LuAG:Ce, even though the latter is more stable in light emission over time. Given the smaller thickness and higher light yield than the selected competitor scintillators, GaGG:Ce was found most suitable under the present experimental conditions.

#### 3.2.3. Convective cooling

The potential for reducing thermal stress in scintillators through convective cooling has previously been explored via simulation (Kastengren, 2019 ▸) and experimentally demonstrated in a standalone context (Bidola *et al.*, 2023 ▸). These studies indicate that convective cooling could mitigate the risk of thermally induced damage and preserve visible light output. Building upon these insights, we implemented a dedicated cooling setup for use within a synchrotron X-ray imaging environment. Specifically, we designed a controlled laminar nitro­gen gas flow (Fig. 4 ▸), both to reduce thermal load and to prevent dust accumulation on the scintillator surface due to electrostatic attraction (Zhou *et al.*, 2018 ▸).

To achieve this, a nitro­gen tank positioned outside the experimental hutch provided a steady gas stream at 1.5 bar, effectively cooling the scintillator during synchrotron irradiation. The flooded gas was subsequently extracted via an outlet system. While the cooling principle was previously validated on the scintillator alone (Bidola *et al.*, 2023 ▸), our study integrates this approach into an operational synchrotron imaging system and assesses its performance under imaging conditions. For this test, the white X-ray beam was attenuated using a 48 mm thick aluminium filter, as illustrated schematically in Fig. 4 ▸.

**Figure 6:**
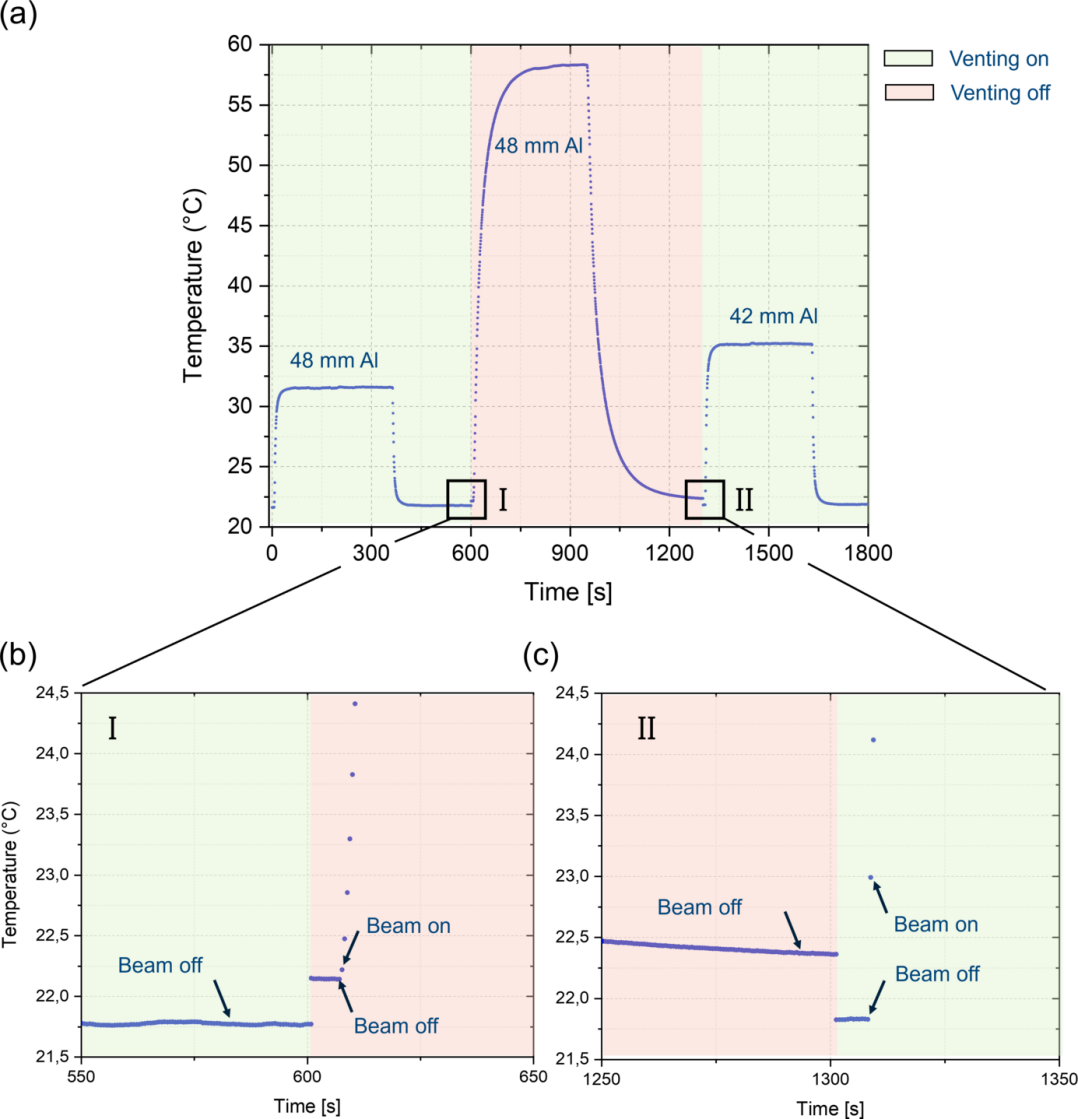
(*a*) Temperature response of the scintillator with a 48 mm Al attenuator. The ascending curve corresponds to heating during exposure and the descending curve to cooling after beam cessation. Green shading indicates active convective cooling by ventilation, whereas red shading indicates measurements without active cooling. The steady-state temperature for each exposure is referenced to the respective attenuator thickness. Composite measurements are shown, with regions I and II enlarged in panels (*b*) and (*c*). (*b*) Comparison of cooled and uncooled conditions, highlighting the temperature gradient and the slower rise without cooling. (*c*) Measurement with a thinner attenuator (42 mm), showing a higher equilibrium temperature and a more rapid temperature rise compared with 48 mm.

The temperature variation near the point of impact of the beam on the scintillator is measured as a function of time with a Keithley Pt100 sensor, and in two stages, which we will call segments: (i) exposure of the scintillator until the steady state is reached, and (ii) cessation of exposure until the state of thermal equilibrium. This process is carried out once with active convective cooling and once without it. A sequence of these measurements in a graph is created in the first two segments of Fig. 6 ▸(*a*).

Fig. 6 ▸ shows that the temperature rise is significantly reduced and the equilibrium state reached more quickly with active convective cooling. With a thinner aluminium attenuator (42 mm), the steady-state temperature during exposure is higher than with a 48 mm attenuator, indicating that pre-hardening the radiation reduces the thermal load on the scintillator, while fewer attenuators increase the demand for efficient cooling. The Pt100 sensor measures the local surface temperature near the beam footprint; although this does not capture the bulk temperature, the rise and decay are representative of the overall thermal load on the scintillator and thus of the induced thermal stress. While a direct correlation with light yield was not established here, previous work (Bidola *et al.*, 2023 ▸) has shown that convective cooling preserves scintillator output under comparable conditions. Taken together, these results indicate that convective cooling substantially reduces the thermal load on the scintillator and thereby mitigates the risk of heat-induced fracture, particularly when the beam is less attenuated.

The intersections of the segments denoted by (I) and (II) in Fig. 6 ▸(*a*) are enlarged in Figs. 6 ▸(*b*) and 6 ▸(*c*), respectively. For a scintillator that has not been exposed to X-rays, the effect of active convective cooling of the scintillator is illustrated by the temperature gradient in Figs. 6 ▸(*b*) and 6 ▸(*c*). In addition, Fig. 6 ▸(*c*) shows a comparatively faster rise in temperature to steady state when convection cooling is applied.

#### 3.2.4. Diamond plate

More efficient scintillator cooling is required, in addition to active convective cooling, for intense radiation or thin attenuators. Considering the transparency of diamond plates to X-rays and the material’s ability to dissipate heat (Kandlakunta *et al.*, 2019 ▸), the mount for the scintillator was configured such that (i) the X-rays first pass through a diamond plate before reaching the scintillator, and (ii) convective cooling is applied through venting directly to one side of the scintillator.

**Figure 7:**
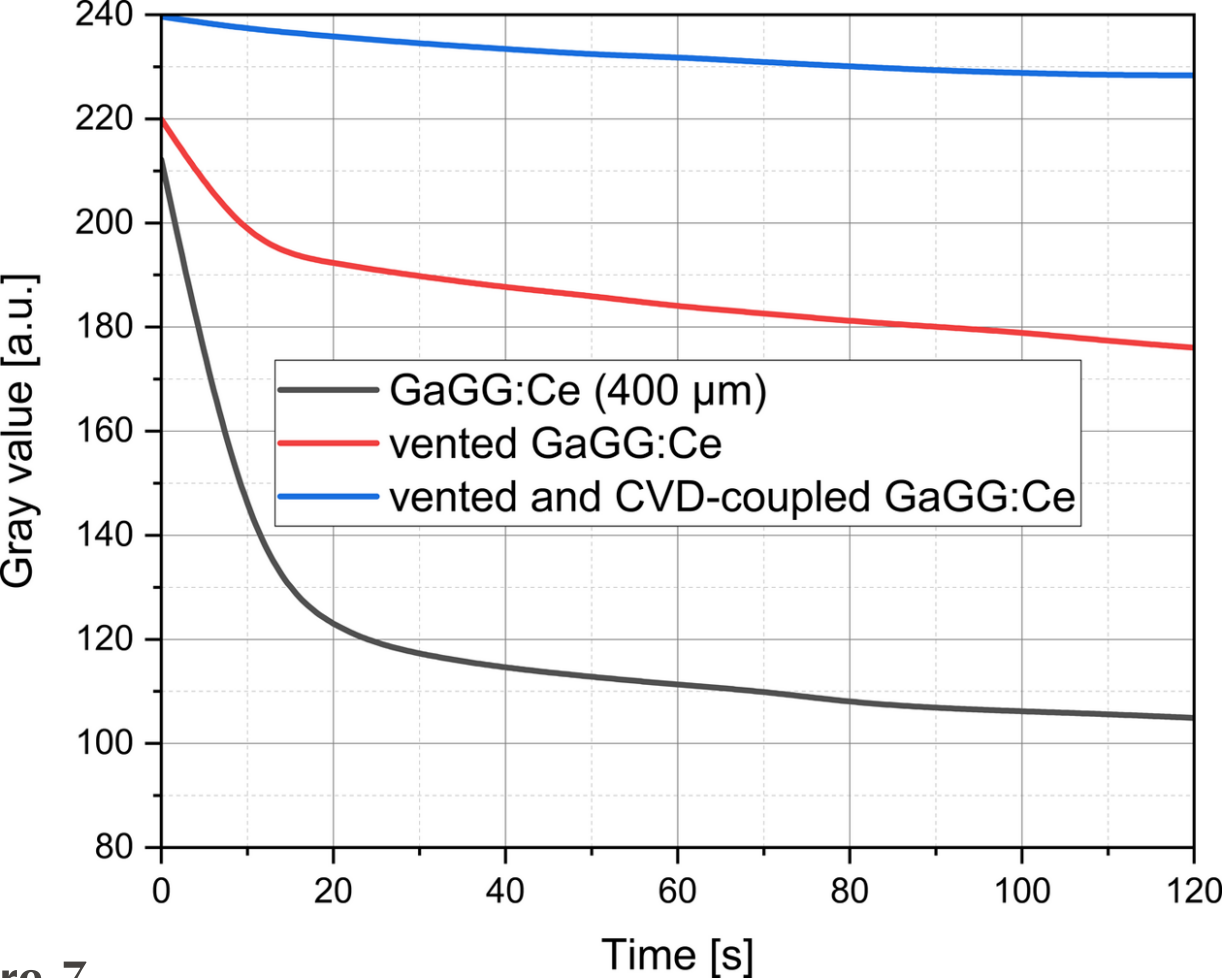
Benefit of additional coupling of the scintillator to a diamond plate to reduce heat stress in a scintillator exposed to the intense white X-ray beam. The plots show the scintillator light output in the form of grayscale values measured at 10 s intervals in the same 280 × 240 pixel segment of captured flat-field images. Measurements were performed without cooling, with active convective cooling and with active convective cooling combined with coupling to a 100 µm diamond plate.

In Fig. 7 ▸, a representative area of 240 × 284 pixels exposed to the white beam is shown to illustrate the response of a 400 µm thick GaGG:Ce scintillator under three different cooling scenarios. Comparative 8-bit images were recorded for a non-vented configuration (dark curve), a vented configuration (red curve) and a vented configuration coupled to an additional 100 µm thick polycrystalline CVD diamond plate (blue curve). For each case, the mean grayscale value of the representative area was computed from images collected every 10 s. The slower decrease in grayscale values observed when the scintillator was both vented and coupled to the diamond plate demonstrates the increased efficiency of the diamond in dissipating heat. This effect is particularly important since thermal stress reduces the light output of the scintillator, and consequently the grayscale intensity recorded by the camera.

#### 3.2.5. Spatial resolution

**Figure 8:**
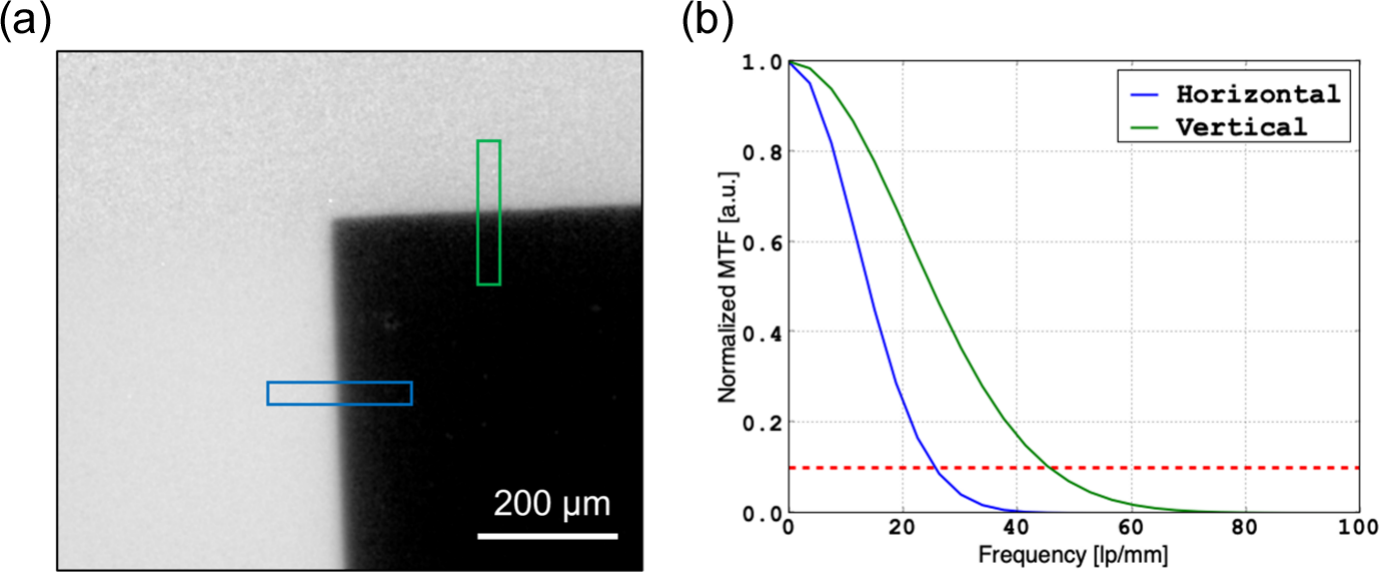
(*a*) Flat-field corrected X-ray image of a 5 mm thick tungsten plate mounted in proximity to the scintillator frame. The areas marked in blue and green at the edges of the tungsten plate are used to evaluate the resolution using the modulation transfer function. (*b*) Modulation transfer function for the horizontal and vertical directions (blue and green curves, respectively).

The spatial resolution is determined by calculating the modulated transfer function (MTF). The edge of the 5 mm thick tungsten plate placed directly in front of the scintillator holder was used. The radiograph is captured in binned data acquisition mode using a 10× objective, representing the standard setup we used in our PBF-EB imaging experiments, and resulting in an effective pixel size of 2.7 µm in Fig. 8 ▸(*a*). In each of the regions marked in the horizontal (blue) and vertical (green) directions, respectively, an average of the line profiles was plotted to which an error function was fitted. With the derivative of the error function and a subsequent Fourier transform, the MTF is evaluated as function of frequencies in line pairs (lp) per millimetre [Fig. 8 ▸(*b*)]. The 10% point of the MTF is indicated by the red dotted line in Fig. 8 ▸(*b*) and is referred to as the resolving power of the system (Müller *et al.*, 2017 ▸). It corresponds to Nyquist frequencies of 26.5 lp mm^−1^ and 49.1 lp mm^−1^ in the vertical and horizontal directions, respectively. Consequently, the spatial resolutions corresponding to half the inverted Nyquist frequency amount to ∼10 µm and 19 µm, respectively.

#### 3.2.6. Evaluation of single-distance propagation-based phase contrast imaging

Provided that the synchrotron radiation is partially coherent, it is possible to apply phase imaging in propagation (Rack *et al.*, 2014 ▸), where propagation refers to the sample-to-detector distance (SDD). This can be achieved to a certain extent with a white synchrotron X-ray beam (Agrawal *et al.*, 2021 ▸). Optimization of single-distance phase contrast with white synchrotron X-rays has proved useful for applied fast imaging (Morgan *et al.*, 2019 ▸).

**Figure 9:**
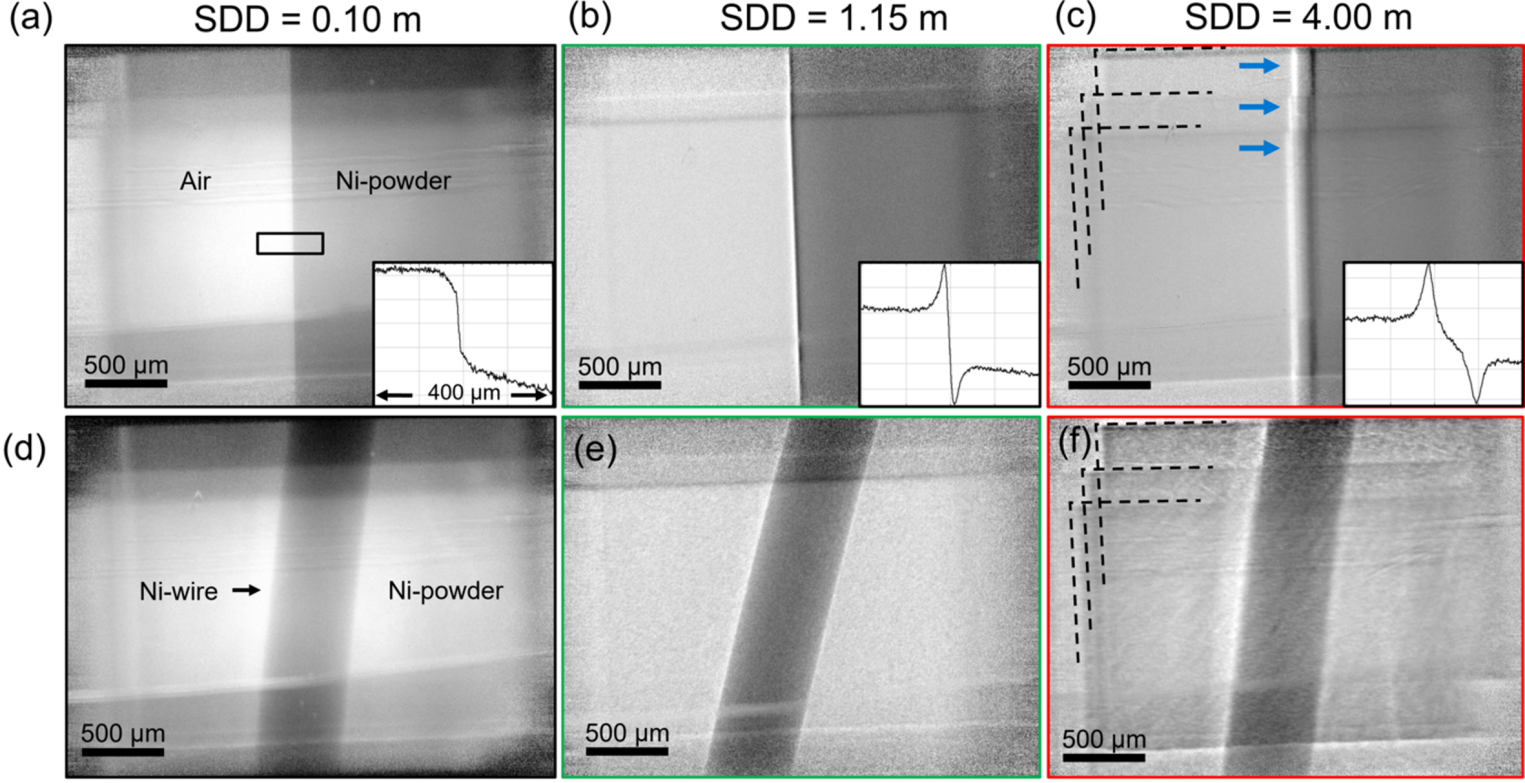
Determining the sample-to-detector distance (SDD) for optimum fringe contrast. The representative sample is an Ni wire of 500 µm diameter embedded in a polystyrene container filled with Ni powder. (*a*) Flat-field corrected projection of the edge of the container. This image is considered pure absorption as it was taken from a short SDD of 0.1 m. (*b*) Projection at an SDD of 1.15 m, showing edge enhancement at the interface. (*c*) Projection at an SDD of 4 m. A profile of the region of interest (ROI) marked in panel (*a*) is displayed in panels (*a*)–(*c*). (*d*) Flat-field corrected projection of the Ni wire embedded in Ni powder in the container at an SDD of 0.1 m. (*e*)–(*f*) Images at SDDs of 1.15 and 4 m, respectively. The dashed lines in panels (*c*) and (*f*) indicate the superimposed beams of some of the ten wigglers. The arrows in (*c*) indicate the resolved front and rear edges of the cubic container, illuminated by each of the superimposed beams.

With partial radiation coherence and a high-resolution system capable of detecting fringes, the optimization of the distance for PB-PCI allows superior fringe visibility and contrast of the edge at the interface of two features with very similar absorption coefficients. This is due to the phase shift developing with distance. Partial coherence is favored on P61A by the long beam path of 76 m from the last wiggler to the earliest sample mounting position possible in the experimental hutch. A representative sample consisting of a 500 µm thick Ni wire embedded in a cubic polystyrene container filled with Ni powder was used. Fig. 9 ▸(*a*) shows a flat-field corrected projection of the part of sample containing only the Ni powder at an SDD of 0.1 m. The image at this distance can be described as absorption dominated, as the effect of the phase shift is not yet pronounced, which is shown by the profile of the marked region of interest (ROI). At an SDD of 1.15 m in Fig. 9 ▸(*b*), the fringes at the air–container interface are visible, as showed by the Laplacian-like profile of the ROI. At an SDD of 4 m, the profile of the ROI shows a differential-like course, which is caused by the more pronounced rear edge of the tilted cubic container. At this distance, the contributions to the beam from some of the ten consecutive wigglers (dashed lines in Fig. 9 ▸) lead to multiple edges of the container [arrows in Fig. 9 ▸(*c*)].

The observations in Figs. 9 ▸(*a*)–9 ▸(*c*) are supported in Figs. 9 ▸(*d*)–9 ▸(*f*), where the Ni wire embedded in Ni powder is displayed. The phase-shift at an SDD of 4 m can be perceived as severe blurring at the interface between the Ni wire and the powder.

The partial coherence of the polychromatic X-rays, the resolution achieved by the imaging system and the evaluation of the propagation distance show that the setup meets the requirements for PB-PCI. With no other rigorous optimization, propagation distances between the MiniMelt and the imaging system of less than 140 cm are established to perform single-distance PB-PCI and avoid blurring from the radiation source at large propagation distances (Gradl *et al.*, 2017 ▸). At large SDDs of about 4 m, artifacts arising from the contributions of multiple wigglers are discussed in more detail in the supporting information. The FoV of 2.7 mm × 2.6 mm is provided by the camera and is reduced to approximately 2 mm × 1.5 mm when the overlapping contributions of the wiggler sources are excluded, which was adopted for the experiments.

## 4. Results and discussion

### 4.1. Thermal management and resolution stability

The coupling of the scintillator with a diamond plate (Fig. 1 ▸) represents a practical enhancement for thermal management in high-speed X-ray imaging systems. This coupling takes advantage of the diamond plate’s high thermal conductivity to dissipate heat and reduce thermal gradients across the scintillator. This configuration supports a stable light output under the high radiation flux conditions of a synchrotron white beam. The dependency of the scintillator light yield on temperature, as demonstrated in Fig. 7 ▸, underscores this advantage. Without coupling, thermal quenching results in a more rapid decline in light yield. By contrast, coupling with the diamond plate ensures steadier and higher light output, demonstrating its role in maintaining optical performance under extreme conditions.

The integration of nitro­gen gas cooling in combination with the diamond plate enhances the effectiveness of thermal management. This dual approach not only prevents heat-induced degradation but also ensures reliable operation during prolonged experiments. These thermal management strategies do not compromise spatial resolution, as shown by the MTF analysis in Fig. 8 ▸. The system maintains resolution values consistent with its design specifications, indicating that the thermal management measures are compatible with the performance of the optical system.

### 4.2. Scintillator optimization

The choice of GaGG:Ce scintillators in this study reflects an optimized selection of optical components for high-speed X-ray imaging. Fig. 5 ▸ shows that GaGG:Ce exhibits a higher light yield, good radiation hardness and an efficient conversion of X-ray energy into visible light, which is well suited for the employed high-speed camera. The high light yield of GaGG:Ce enhances the signal-to-noise ratio, an important factor for capturing fine details in rapid imaging applications. Its radiation hardness enables the scintillator to withstand prolonged exposure to intense radiation flux without significant degradation, maintaining consistent optical performance over extended experimental durations (Park *et al.*, 2024 ▸). By producing bright and stable visible light in proportion to the deposited X-ray energy, GaGG:Ce facilitates high-quality image acquisition even at rapid frame rates. These attributes demonstrate that GaGG:Ce is a suitable choice for high-flux synchrotron imaging applications. Recent work by Hwang *et al.* (2025 ▸) on the PLS-II 6C beamline compared LuAG:Ce and GaGG:Ce scintillators under monochromatic X-ray illumination (20 keV) and confirmed the superior light yield and signal-to-noise characteristics of GaGG:Ce. Although their study was performed at lower flux and without white beam exposure, their conclusions align with our findings that GaGG:Ce provides excellent optical efficiency and stability, making it well suited for high-speed synchrotron imaging applications.

### 4.3. Single-distance propagation-based imaging

The implementation of single-distance PB-PCI illustrates the system’s optical performance. As shown in Fig. 9 ▸, optimization of the SDD is critical for enhancing phase effects and achieving a high fringe contrast. This effect originates from Fresnel (near-field) diffraction, which governs the propagation of X-rays after passing through the sample. At shorter SDDs, imaging primarily captures absorption, resulting in less pronounced edges. However, as the SDD increases (*e.g.* 1.15 m), near-field phase shifts at material interfaces produce pronounced fringe contrasts, improving the visibility of fine structural details. Figs. S2 and S3 highlight the interplay between propagation distance and image artifacts. While longer SDDs can improve phase contrast, they also introduce artifacts due to the superposition of beam sources from multiple wigglers. These artifacts, including duplicated features, compromise image integrity. Limiting the effective propagation distance to approximately 1.4 m minimizes these artifacts while preserving phase contrast effects.

The ability to achieve high-resolution PB-PCI with partially coherent white synchrotron X-rays confirms that the setup provides sufficient resolving power and sensitivity to phase variations. The careful design of the optical path, inclusion of high-pass filters and use of GaGG:Ce scintillators together enable the visualization of features with minimal absorption contrast but significant phase shifts. These findings highlight the versatility of the system for resolving fine structural features in phase contrast imaging applications.

### 4.4. Crack monitoring during PBF-EB of CMSX-4

The capability of the presented imaging setup to monitor cracking under realistic PBF-EB processing conditions was evaluated during the processing of CMSX-4 powder. CMSX-4 is a well known second-generation single-crystal Ni-based superalloy, characterized by high concentrations of tungsten, tantalum and rhenium, which make it particularly challenging to perform imaging experiments with (the full composition is provided in Table 1 ▸). For crack-free PBF-EB processing, CMSX-4 typically requires a processing temperature above 1000°C (Bäreis *et al.*, 2023 ▸) with minimal temperature fluctuations to mitigate thermal stresses.

Using the MiniMelt system, CMSX-4 powder was processed in build experiments comprising several hundred layers. A single melt track, approximately 1 mm thick and 15 mm long, was generated, with the melting direction oriented perpendicular to the synchrotron beam. A layer height of 50 µm was maintained. The process began at a build temperature of 1050°C and gradually cooled until cracks formed. To ensure consistent powder bed and sample temperatures while achieving a material volume thin enough for imaging, the melt track was created atop a trapezoidal powder hill. At the imaging location, the total sample thickness, including the solid melt track and surrounding powder, was estimated to be approximately 4 mm.

The scintillator assembly used in the experiment comprised a 400 µm thick GaGG:Ce scintillator and a 100 µm thick diamond plate. Cooling of the assembly was achieved solely through convective cooling using N_2_ gas. An optical magnification of 10× was employed and the camera was operated in high-speed mode with 2× binning (HS binned), resulting in an effective pixel size of 2.7 µm. The experiments were conducted at PETRA III using the 40-bunch time-resolved mode. Pre­liminary tests with CMSX-4 powder revealed that the unatten­uated synchrotron beam caused significant heat load, partially melting the powder. To mitigate this, a 15 mm thick Al absorber was placed in the synchrotron beam path before the sample. This not only reduced the heat load on the powder but also protected the scintillator, which could not withstand the unattenuated beam.

The SDD was set to 120 cm. Data collection during processing was automatically triggered by the MiniMelt system, ensuring a consistent delay of 1 to 2 ms between triggering and data acquisition. Imaging was performed at an acquisition rate of 6 kHz with an exposure time of 160 µs.

As shown in the contour plot of the critical propagation distance in Fig. 2 of the report by Mayo *et al.* (2012 ▸), the propagation distance is well below the critical value, with the photon energy at this distance being approximately 50 keV. This indicates that the energy range of 40 to 70 keV in Fig. 3 ▸(*b*) plays a significant role in contributing to the fringe contrast, despite the spectral weighted average energy of the spectrum after the filters exceeding 100 keV (see Table S2 in the supporting information).

**Figure 10:**
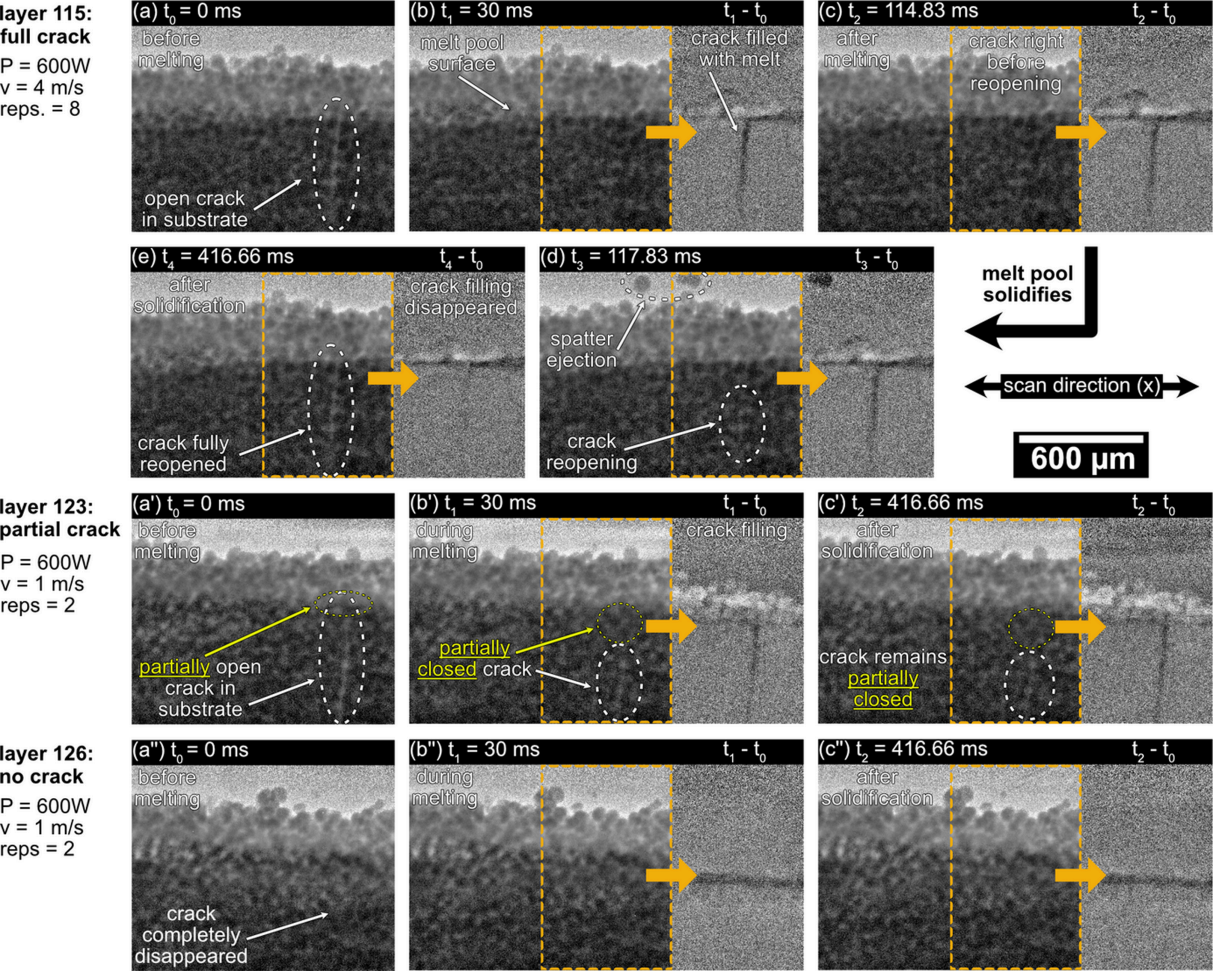
Evolution of a crack during melting of CMSX-4 powder over multiple layers. (*a*)–(*e*) A fully open crack that gets closed during melting, followed by a complete reopening during solidification. The release of tension during crack reopening induces spatter particle ejection [panel (*d*)]. (*a*′)–(*c*′) A partially closed crack is further closed during melting and remains in that partially closed state after solidification. (*a*′′)–(*c*′′) The crack has fully disappeared and does not reappear upon melting and solidification. For each layer shown, a video of the full layer melting procedure is provided in the supporting information.

An example of crack monitoring captured during the powder melting experiments is presented in Fig. 10 ▸, illustrating various stages of crack evolution over 11 layers. The displayed images were flat-field corrected and had their contrast enhanced using contrast-limited adaptive histogram equalization (CLAHE). To emphasize crack evolution further, difference images were created by subtracting the first frame of the recording from subsequent frames (*t*_i_ − *t*_0_).

The crack was initially observed at layer 114, when the build process temperature (measured underneath the build plate) had dropped to approximately 900°C. The image series begins with a fully opened crack in Fig. 10 ▸(*a*) prior to melting in layer 115, followed by complete crack closure during melting [Fig. 10 ▸(*b*)] and reopening during solidification [Figs. 10 ▸(*c*)–10 ▸(*e*)]. The reopening is accompanied by tension release, causing sufficient surface movement to eject two powder particles from the powder bed, as seen in Fig. 10 ▸(*d*).

The imaging system revealed a progressive reduction in crack size with subsequent layers, underscoring the role of thermal cycling, the use of melting parameters and layer consolidation in mitigating defect propagation. This understanding can inform strategies to process crack-sensitive materials like Ni-based superalloys more effectively. By layer 122, the crack starts in a partially closed state in Fig. 10 ▸(*a*′) and undergoes further closure during melting [Fig. 10 ▸(*b*′)]. Notably, the crack no longer reopens during solidification in Fig. 10 ▸(*c*′). After three more layers, the crack is eliminated and does not reappear during subsequent remelting or solidification [Figs. 10 ▸(*a*′′)–10 ▸(*c*′′).

To our knowledge, this multi-stage crack formation and closing behavior in PBF-EB has not been previously reported and it illustrates the potential of high-speed *in situ* imaging for studying such dynamic phenomena.

The presented results allow the identification of key characteristics of crack formation when processing CMSX-4 powder via PBF-EB. First, no cracking was observed before layer 114 at the initially higher processing temperatures (starting from 1050°C at the beginning of the experiment). This shows that a temperature threshold is needed before cracking starts to occur and that this temperature threshold depends on the sample geometry, as cracking of CMSX-4 in PBF-EB has been observed at significantly higher temperatures (>1000°C) for larger sample sizes (Bäreis *et al.*, 2023 ▸). Second, the timing of crack formation in the recorded image series in Fig. 10 ▸ allows us to infer that cracking is most likely induced through the accumulation of residual stresses. This means that cold cracking is most likely to be responsible for crack formation, and not hot cracking mechanisms like solidification or liquation cracking, which would happen faster during the solidification process and not after it has finished (as is the case in the images shown here). The onset of crack formation below a certain temperature threshold further supports this conclusion, as solidification and liquation cracking would be observable at higher processing temperatures as well. Finally, the recorded cracking phenomena show that cracks can be closed during the process, given adequate melting conditions. In the image series shown here, the interaction time between the electron beam and the powder bed, as introduced by Körner *et al.* (2013 ▸) and Semjatov *et al.* (2024 ▸), was increased across the 11 observed layers and was probably a contributing factor in the eventual closure of the crack. In layer 115 from Figs. 10 ▸(*a*)–10 ▸(*e*), melting took place at a scan velocity of 4 m s^−1^, resulting in a beam interaction time of 185 µs (given an electron beam spot size of 740 µm *D*4σ), whereas a scan velocity of only 1 m s^−1^ and subsequently a beam interaction time of 740 µs were used from layer 123 onwards. As higher beam interaction times have been shown to lead to larger melt pool depths (Semjatov *et al.*, 2024 ▸), given the same beam power was used, the closing of the crack was most likely induced by the creation of a deeper melt pool, starting with layer 123 where the first partial closure of the crack was observed.

This example demonstrates that by tracking crack progression across 11 layers in real time, it was possible to identify several key characteristics of crack formation for the chosen material that would have been difficult to observe otherwise. While it is possible to observe the onset of crack formation with varying processing temperature using other process monitoring techniques like electron optical imaging [like was done by Bäreis *et al.* (2023 ▸)], the identification of the cracking mechanism itself and the effect of small changes in processing strategy on the cracking behavior would have remained elusive without the use of high-speed *in situ*X-ray imaging. As the identification of the cracking mechanism, be it hot or cold cracking, demands high temporal resolution, the monitoring of crack depth or partial crack closure would be impossible without high spatial resolution and the ability to penetrate deeply into the sample material offered by white beam synchrotron imaging. This capability provides insights into long-term material performance in additive manufacturing. The quality of the imaging data allows a clear distinction between different stages of crack evolution, enabling investigations into how cracking is influenced by processing conditions and melting parameters.

In the case discussed here, the synchrotron beam required substantial attenuation with an aluminium absorber to prevent heating and melting of the CMSX-4 powder. At 40 m from the source, the P61 high-energy wiggler beamline produces a beam with a nominal power density of ∼121 W mm^−2^. The filtered incident power reaching the sample, combined with the high X-ray absorbance and low thermal conductivity of CMSX-4 powder, is sufficient to cause local melting under illumination at this synchrotron source. This also indicates that significantly more flux would be available for less absorbent materials prone to cracking, as the flux was strongly reduced by the absorber.

*In situ* imaging of crack formation during powder bed fusion has been previously reported, notably by Rollett and co-workers at the Advanced Photon Source (USA) using dynamic X-ray radiography (Chiang *et al.*, 2019 ▸). However, these investigations typically involve relatively thin sample thicknesses of the order of a few hundred micrometres, which limits their applicability to thicker geometries. To our knowledge, crack formation during powder bed fusion has not yet been monitored *in situ* at the larger sample thicknesses studied here. For comparison, Ghasemi-Tabasi *et al.* (2022 ▸) examined CM247LC (which has an absorptivity similar to CMSX-4) at a 10 kHz acquisition rate, but only with effective thicknesses of a few hundred micrometres detected under PBF-LB conditions, unlike PBF-EB which requires pre-sintered powder beds for stable operation.

#### 4.4.1. Outlook for optical system design

Single-distance phase contrast imaging has been demonstrated to enhance crack visualization in materials by providing improved contrast compared with traditional absorption-based methods. This technique is particularly effective in detecting features such as voids, porosity and boundaries between similar materials, which are essential for studying cracks and other material failure mechanisms. For instance, in the investigation of Al:SiC metal matrix composites, phase contrast imaging has been utilized to identify the rupture of SiC particles as the initiation point of plastic deformation (Mayo *et al.*, 2012 ▸). Additionally, phase contrast X-ray imaging has been applied to observe solidification and hot crack formation in laser manufacturing, providing better understanding of these processes (Miyagi *et al.*, 2018 ▸). These studies highlight the utility of single-distance phase contrast imaging in enhancing the visualization of cracks in various materials, offering valuable insights into material behavior and failure mechanisms.

The findings of this study may have broader relevance to the development of high-speed high-resolution imaging systems. These advances can inspire new methodologies in synchrotron-based imaging and beyond, paving the way for applications in laser welding, dynamic process monitoring and other high-energy environments.

By combining robust thermal management with precise optical design, this work provides a framework for future innovations in optical imaging technologies. The strategic integration of advanced materials and optimized optical pathways underscores the potential for continued exploration and refinement of high-speed imaging systems to address emerging challenges in materials science and beyond.

To our knowledge, comparable high-speed X-ray imaging systems that enable *in situ* monitoring of cracking during realistic PBF-EB processing conditions have not yet been reported.

Fig. 10 ▸ highlights the effectiveness of a high-resolution imaging system designed for extreme conditions and its application to studying ultrafast phenomena like melt pool and material evolution in PBF-EB. The very high flux delivered to the experimental chamber in this work was carefully managed using attenuators and filters, protecting the equipment from radiation damage and improving image contrast (Fig. 3 ▸). This explains the selection of different filter combinations for the results shown in Fig. 10 ▸. Exploring additional filter options, such as Zr–W matrices (Di Michiel *et al.*, 2005 ▸) and Sigradu (Marone *et al.*, 2020 ▸), could broaden the system’s applicability for various experiments.

The selection of scintillators compatible with the beamline (Fig. 5 ▸) was critical to the performance of the imaging system. The integration of an active convection cooling system (Fig. 6 ▸) effectively preserved the scintillator’s light emission efficiency during high-speed imaging, mitigating the thermal stress induced by intense radiation. From an optimization standpoint, investigating alternative scintillators with shorter decay times, such as LYSO:Ce, is recommended. These materials could offer higher light yield and dynamic range, particularly in PETRA III’s time-resolved mode (40- and 60-bunch) (Rutherford *et al.*, 2016 ▸; Pidol *et al.*, 2004 ▸), while also better aligning the scintillator’s properties with the numerical aperture (NA) of the optical system (Xie *et al.*, 2016 ▸; Xie *et al.*, 2019 ▸). It should be noted that the current system is optimized for visible light emitting scintillators (≥400 nm) and is therefore not well suited for UV-emitting materials, as the camera quantum efficiency decreases sharply below this range.

The enhanced heat dissipation allowed the scintillator to maintain sufficient light yield during prolonged measurements (König *et al.*, 2023 ▸; Ye *et al.*, 2024 ▸). Consequently, the design outlined in Fig. 1 ▸(*c*) could benefit other beamlines facing radiation-induced image degradation. While the setup supported ultrafast imaging at 15 kHz (König *et al.*, 2023 ▸), a slight variation in scintillator light emission was observed during experiments, as shown in Fig. 7 ▸. This fluctuation can compromise flat-field correction and potentially lead to misinterpretations. These observations indicate the importance of further advances, such as improved cooling solutions for scintillators, the use of a fast shutter or the implementation of advanced post-processing techniques to address these challenges.

## 5. Conclusion

We designed and characterized an X-ray microscope optimized for high-flux operations on the P61 beamline at DESY. The setup was tailored for *in situ* high-speed white beam imaging of the PBF-EB process. The tiered arrangement of mirrors for guiding light from the scintillator-converted white beam (Halls *et al.*, 2017 ▸) and the use of thick shielding (Wroblewski, 2015 ▸) effectively reduced the presence of high-energy diffracted photons (‘zingers’) that can interfere with the camera sensor and degrade image quality.

We demonstrated the advantages of employing a dedicated nitro­gen gas stream to cool the scintillator uniformly. Combined with coupling the scintillator to a diamond plate, these measures were key to improving the scintillator’s durability and light yield.

The flux and heat load on all beam path components were minimized using filters and absorbers. Suitable scintillators for specific applications and environmental conditions were carefully evaluated (Di Michiel *et al.*, 2005 ▸). The final design also enabled single-distance PB-PCI due to the partial coherence provided by the synchrotron white beam radiation. A critical propagation distance of approximately 140 cm between the scintillator and the camera was observed, consistent with predictions from Weitkamp *et al.* (2011 ▸) for single-distance phase imaging.

Beyond PBF-EB, the system’s capability for ultrafast imaging and real-time defect monitoring makes it a promising tool for studying dynamic processes in laser welding (Kaufmann *et al.*, 2023 ▸; Hollatz *et al.*, 2022 ▸; Schricker *et al.*, 2023 ▸), cavity fracture and collapse studies (Hollatz *et al.*, 2022 ▸), and other industrial applications requiring high spatial and temporal resolution. Additionally, it holds potential for dynamic single-shot phase contrast imaging methods involving gratings (Rack *et al.*, 2024 ▸), particularly Talbot illuminators (Gustschin *et al.*, 2021 ▸), with appropriate adaptations to withstand the beamline’s flux and align with its spectral energy range.

The ability of this X-ray imaging system to capture real-time high-resolution data on crack formation and evolution in challenging materials like CMSX-4 represents an important step forward for additive manufacturing research. By providing actionable insights into process–structure relationships, this system not only supports optimization of PBF-EB parameters but also establishes a basis for addressing long-standing challenges in processing crack-sensitive materials. Its adaptability to other dynamic processes, coupled with its innovative optical design, make it a versatile tool for supporting advances in both additive manufacturing and high-speed imaging techniques across a range of disciplines.

## Supplementary Material

Sections S1 to S2 including Figures S1 to S4 and Tables S1 to S2. DOI: 10.1107/S1600577525010057/mo5309sup1.pdf

Layer 115 - Full crack evolution. DOI: 10.1107/S1600577525010057/mo5309sup2.mp4

Layer 123 - Partial crack evolution. DOI: 10.1107/S1600577525010057/mo5309sup3.mp4

Layer 126 - No crack. DOI: 10.1107/S1600577525010057/mo5309sup4.mp4

## Figures and Tables

**Table 1:** Alloy composition of the CMSX-4 powder used for single-line track melting experiments (values in wt%)

Ni	Co	Cr	Ta	W	Al	Re	Ti	Mo	Hf
Bal.	9.93	6.6	6.57	6.44	6.15	2.91	1.08	0.65	0.085

## Data Availability

Data underlying the results presented in this paper may be obtained from the authors upon reasonable request.
