# Supplementary material for: Time-resolved imaging of electron beam powder bed fusion using an X-ray microscope optimized for white beam radiation
Source: J Synchrotron Radiat. 2026 Jan 1;33(Pt 1):181–94. doi: 10.1107/S1600577525010057 (PMC12809454; doi:10.1107/S1600577525010057)
Supplement: Supplementary file 1 [file s-33-00181-sup1.pdf]

JOURNAL OF SYNCHROTRON RADIATION

Volume 33 (2026)

Supporting information for article:

Time-resolved imaging of electron beam powder bed fusion using an X-ray microscope optimized for white beam radiation

Pidassa Malimda Bidola, Pidassa Bidola, Nick Semjatov, Gabriel Spartacus, Hans-Henrik König, Guilherme Abreu-Faria, Johannes Klingenberg, Jens Brehling, Christina Krywka, Peter Staron, Greta Lindwall, Carolin Körner, Chrysoula Ioannidou and Felix Beckmann

The supplementary document provides details on the materials used in the simulation, including the electron beam melting (EBM) system. It further describes the influence of the filters on the white beam spectrum and their implications for imaging performance. Additional analyses of fringe contrast in single-distance propagation-based phase contrast imaging (PB-PCI) are included, along with illustrations of setup-related artifacts, that directly affect PB-PCI image quality.

S1. Methods

S1.1. Simulation using SPECTRA

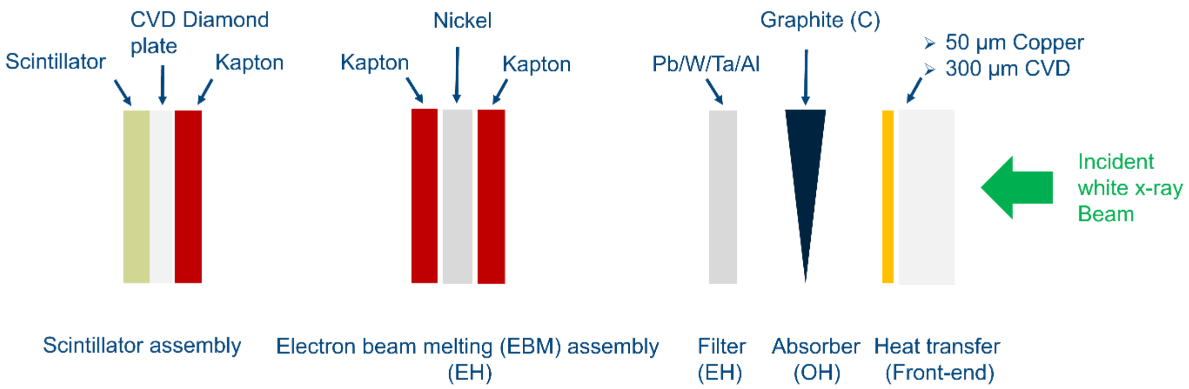

Figure S1 Superposition of the components in the radiation path from the front end to the X-ray machine in the experimental hutch (EH) via the optical hutch.

The white synchrotron radiation spectrum generated by ten consecutive wigglers was simulated using SPECTRA software, applying the machine parameters of the facility (Farla *et al*., 2022). A 50 µm copper plate and a 300 µm CVD diamond layer were included as mandatory components for radiation heat attenuation. An additional graphite absorber located in the optical hutch can be introduced when further attenuation is required. In the experimental hutch, filters composed of various materials (Pb, W, Ta, Al) are positioned immediately upstream of the EBM system. The section of the EBM chamber exposed to radiation comprises two Kapton windows that maintain the vacuum around the nickel sample. For the simulation, a nickel sample thickness of 100 µm and a Kapton window thickness of 200 µm were assumed. The design of the scintillator assembly shown in Fig. 1 illustrates the configuration of the scintillator and the diamond plate implemented in the simulation.

Each material can be added directly to the simulation program as a filter, except for the scintillator, whose composition requires a ratio distribution. Table S1 shows, for example, the ratio calculation for a GaGG scintillator (Gd3Al2Ga3O12).

Table S1 Example of ratio calculation for a GaGG crystal using SPECTRA.

	Gd_3	Al_2	Ga_3	O_{12}
Atomic number	64	13	31	8
Atomic mass	157,25	27	69,72	16
Atomic mass x index	471,75	54	209,16	192
Ratio	0,5089	0,058	0,225	0,207

A subtraction between the spectrum before and after the scintillator represents the spectrum likely to be converted into visible light and is displayed in Fig. 3(b). This spectrum can be modified depending on the selected filter material beforehand and allows therefore a prediction of the expected spectrum and averaged spectral weighted energy.

S1.2. Influence of the filters on the polychromatic spectrum

As the spectrum is simulated as a function of the thickness of the reference material, i.e. 18 mm Al at the scintillator's non-damaging point, the risk of damaging it is reduced when the thickness of another filter or a combination of filters results in a comparatively lower spectral intensity, see Fig. 3(a).

The filter materials used in this work are therefore selected based on their atomic number and hence their ability to dampen high energies. Nevertheless, their melting temperature is essential to withstand the thermal load induced by the beam. The only exception is Pb, while Ta, W and Pt have proven to be functional high-pass filters under current experimental conditions.

Furthermore, the choice of filters has a predominant effect on the energy spectrum of radiation and, consequently, on the contrast of the image after attenuation by the object. This effect of filters on the polychromatic spectrum is expressed, for example, by the spectral weighted average:

$$E_{av} = \frac{\int E\phi(E)dE}{\int \phi(E)dE},$$

where $\phi(E)$ is the photon fluence and E the energy component of the spectrum.

Table S2 shows the E_{av} of the spectrum after transmission of the beam through a few materials. The beam is hardened after each material, shifting the average energy to high energy levels above 100 keV.

According to the simulation, the average energies after the EBM environment using the high-pass filters selected in this work are very similar, around 106 keV. Under these conditions, the variation in contrast is very much restricted, hence the need for measures such as a fast shutter, which will enable

lower radiation exposures to the scintillator, the use of fewer or thinner filters and consequently a more significant contribution of lower energy levels to achieve better contrast. In the current context, a combination of Al and Ta has been used, and GaGG:Ce has been the preferred scintillator.

Table S2 Spectral weighted average of the of the white beam spectrum after selected filters and the sample.

**Materials in the beam path**	**Averaged energy [keV]**
50 µm Cu, 300 µm CVD	62.9
50 µm Cu, 300 µm CVD 18 mm Al	88.78
50 µm Cu, 300 µm CVD, 200 µm Ta, 10 mm Al	104.37
after 200 µm Ta, 10 mm Al & EBM	106.74
after GaGG:Ce	125.32
absorbed in 400 µm GaGG:Ce	81.85
**Materials including the EBM environment**	
50 µm Cu, 300 µm CVD, 200 µm Ta, 10 mm Al & EBM	106.74
50 µm Cu, 300 µm CVD, 200 µm Ta, 1 mm Fe & EBM	113.43
50 µm Cu, 300 µm CVD, 200 µm W, 10 mm Al & EBM	106.76
50 µm Cu, 300 µm CVD, 200 µm Pt, 10 mm Al & EBM	106.14

S2. Investigation of the single-distance propagation-based phase contrast imaging

S2.1. Demonstration of the fringe contrast

We used the tip of a writing pad to demonstrate very simply the effect of the fringe contrast we are interested in, because of its thermal resilience and the structures inside. For this purpose, we will specifically observe the interfaces on the inside of this object.

Figs. S2(a)-(b) show an absorption-corrected flat-field image for an SDD of 0.3 m and an image with a pronounced additional phase shift for an SDD of 0.50 m. To perceive visually the pronounced fringes at the interface of structures within the object as a function of distance, the derivatives of the images in (a) and (b) are shown in Figs. S2(c) and (d). By comparison, profiles in the areas marked red in (a) and blue in (b) show absorption curves in black and additional phase shift curves in blue. The phase shift that has occurred inside the object is manifest at the interfaces as early as 0.5 m, as indicated by the pointers in Figs. S2(d) and (e).

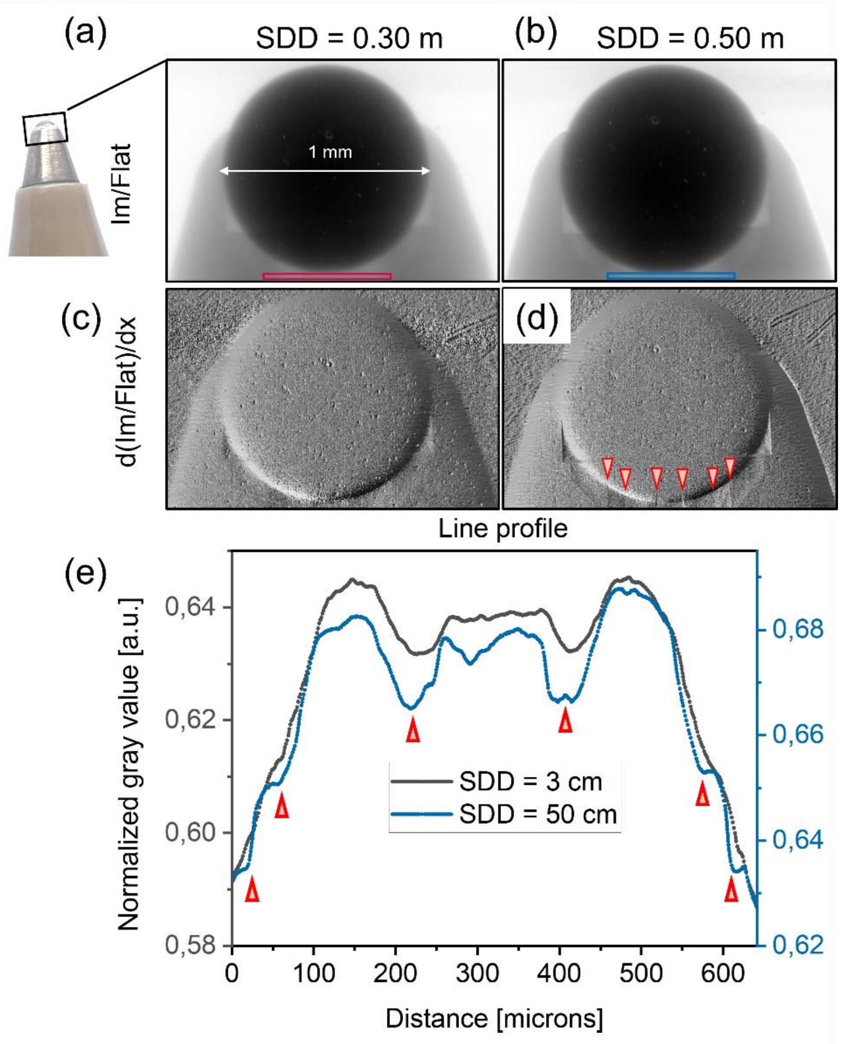

Figure S2 Revealing fringe contrast in single-distance PB-PCI. (a) Flat-field corrected projection of the pen tip. This image is considered absorption as it was taken from a short distance of 0.30 m. (b) Flat-field corrected projection of the sample from 50 cm, showing sharp edges. (c)-(d) Derivatives of images (a) and (b), respectively, giving a visual impression of the phase shift evolved with propagation distance. (e) Profiles of the areas marked in red in (a) and blue in (b). These are shown in black for the absorption and in blue for the image where the phase shift has evolved.

S2.2. Wiggler superposition artifacts

A gold grid for transmission electron microscopy (TEM) has been used to visualize artifacts arising from the relative displacement of multiple wigglers. Figure S3(a) shows a radiograph acquired with a laboratory setup of the gold grid (TEM G300G, VWR International GmbH), which has bar and hole width of 25 and 58 µm, respectively, a thickness of 25 µm, ±5 µm and a diameter of 3.05 mm. Fig. S3(b) presents a radiograph of the gold grid positioned at a SDD of 4 m. The entire field of view (FoV) of the camera of 2.7 mm X 2.6 mm is shown and exhibits at least three superposed source

contributions, indicated by solid arrows. The dashed arrows mark towards a mechanical crack that is not relevant for interpretation. The region outlined in red in the upper window corresponds to illumination from a single wiggler and is detailed in Fig. S3(c). In contrast, the region outlined in blue in the lower part of the FoV, where contributions from at least three wigglers overlap, shows duplication of the gold grid bar width, as highlighted in Fig. S3(d). These results confirm that at long propagation distances, the superposition of multiple wiggler sources produces discernible imaging artifacts.

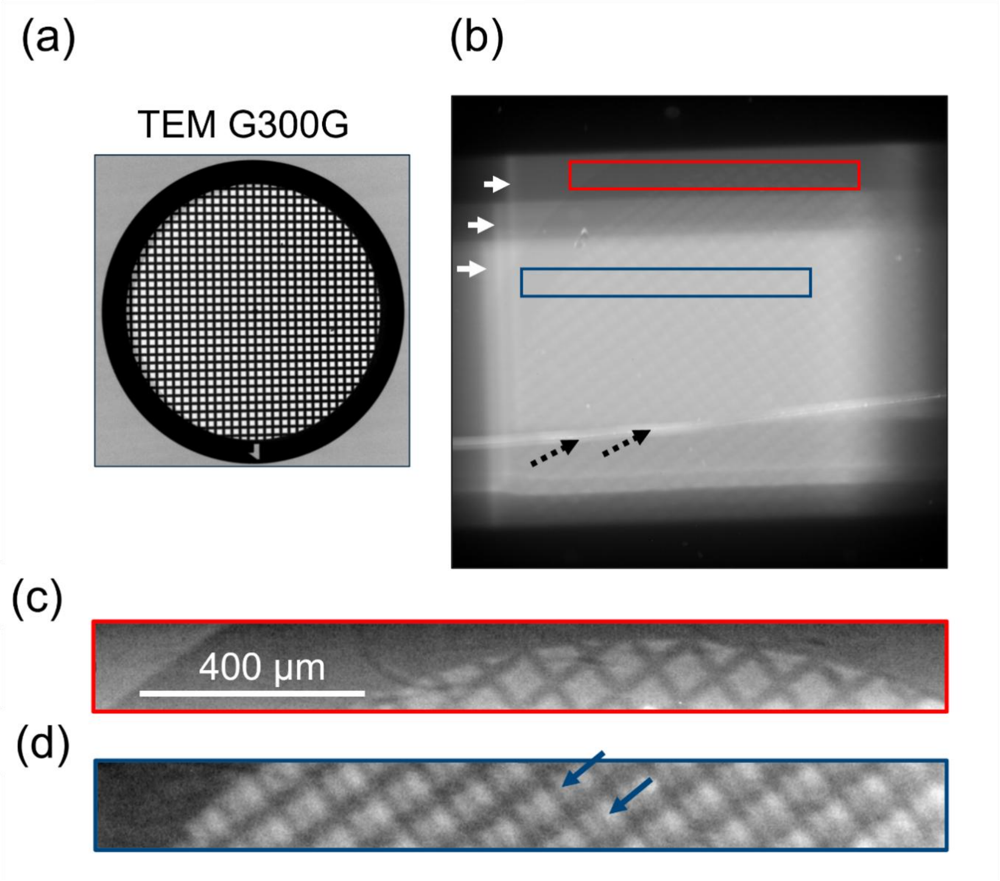

Figure S3 Detecting the superposition of multiple beam sources. (a) Radiograph captured at a lab-source of a gold grid (TEM G300G) with a diameter of 3.05 mm, a thickness of 25 µm, a hole width of 58 µm and a bar width of 25 µm. (b) Projection of the single gold grid mounted 4 m from the X-ray microscope covering the entire FoV of the camera of 2.7 mm x 2.6 mm. The white arrows indicate the perceptible beam shape emanating from multiple wigglers. The dark dashed arrows show a mechanical crack in the scintillator. (c)-(d) Areas marked in red for a single white beam source and blue for superposed sources, respectively. The blue arrows in (d) reveal a replicate of the gold grid bar.

The influence of overlapping wiggler sources is further illustrated in Fig. S4. The visible source regions are delineated from top (green dashed line) to bottom (red dashed line). A bearing was used as an arbitrary sample, positioned at an SDD of 4 m, and incrementally shifted upward. In the initial position (Fig. S4a), the spherical ball within the bearing exhibits sharp edges in the upper window, illuminated by a single source. In the blue-marked region, where two sources overlap, double contours become apparent (solid arrows).. In the lower part of the image, multiple overlapping sources produce several visible edges of the same feature. When the bearing is shifted upward by 300 µm (Fig. S4b), the previously observed double contours (parallel lines in the blue region of Fig. S4a) merge into a single edge, as the feature enters the upper green region illuminated by a single wiggler source. A further upward shift of 500 µm (Fig. S4c) confirms these observations, demonstrating the direct link between feature duplication and the spatial overlap of multiple wiggler sources.

Single-distance PB-PCI could be investigated at long propagation distance up to 4 m using a single wiggler; however, with the current setup, it is constrained by the superposition of multiple sources. Therefore, a propagation distance of approximately 1.40 m is preferred to achieve artifact-free single-distance PB-PCI.

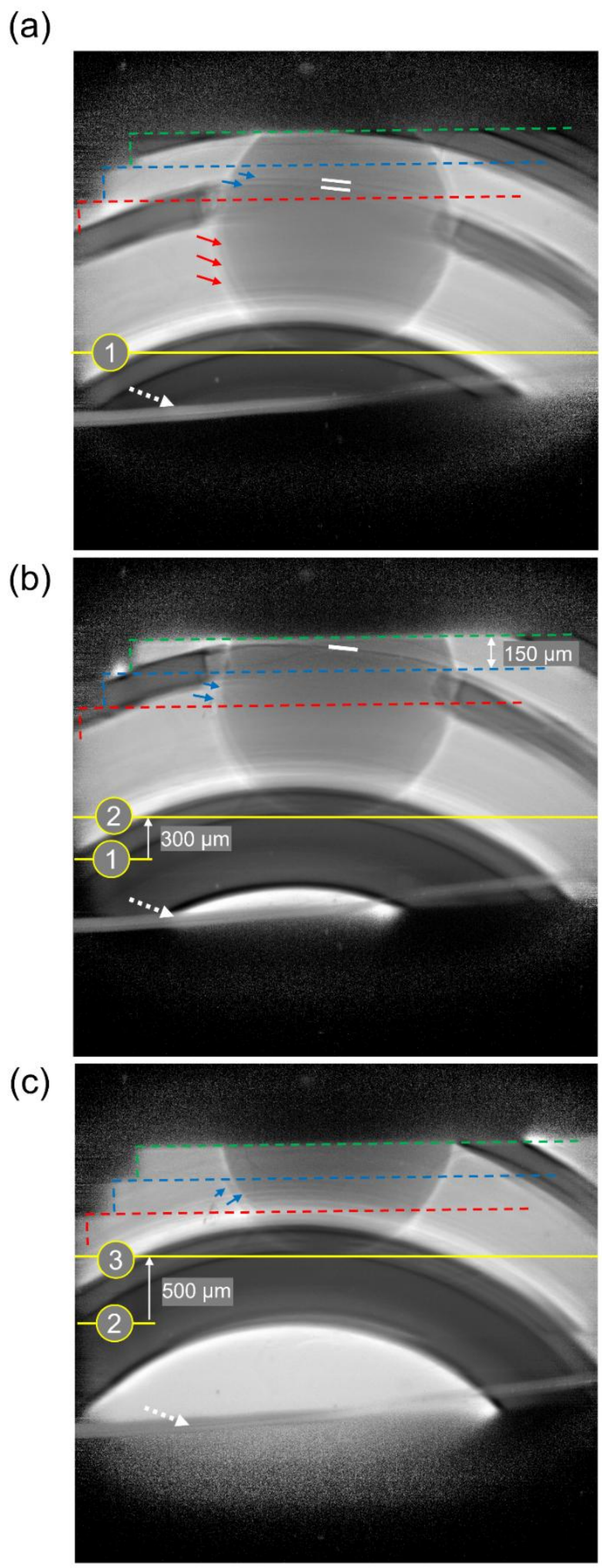

Figure S4 Demonstration of artifacts caused by the superposition of multiple wiggler sources at large propagation distances between the sample and the X-ray microscope. Flat-field corrected radiographs of a bearing that was placed at 4 m from the X-ray microscope and shifted gradually in the vertical direction in the entire FoV of 2.7 mm x 2.6 mm are shown. (a) The superposed wiggler

sources perceptible to the viewer are delimited by dashed colored lines. The upper window reveals sharp edges of a sphere (steel) of a diameter of 1.25 mm. The edges are duplicated which is marked by the arrows in the second window defined in blue. An additional edge is observed in the window delimited in red. The yellow solid line references the initial position of the bearing (1), which will be moved up to replicate the current observations. (b) The bearing is shifted from (1) to (2) about 300 µm and the artifacts are reproduced except in the upper single window. (c) The bearing is moved up about 500 µm from position (2) supporting the artifacts caused by the superposed beams emanating from the consecutive wigglers. The dashed white arrows in the radiographs point to a machinal damage of the scintillator.

References

Farla, R., Bhat, S., Sonntag, S., Chanyshev, A., Ma, S., Ishii, T., Liu, Z., Nishiyama, N., Abreu-Faria, G., Wroblewski, T., Schulte-Schrepping, H., Drube, W., Seeck, O. & Katsura, T. (2022). *J. Synchrotron Rad*. **29**, 409-423.
